# Improving data-driven gated (DDG) PET and CT registration in thoracic lesions: a comparison of AI registration and DDG CT

**DOI:** 10.1186/s40658-025-00797-6

**Published:** 2025-09-30

**Authors:** Tinsu Pan, M. Allan Thomas, Yang Lu, Dershan Luo

**Affiliations:** 1 Department of Imaging Physics, The University of Texas MD Anderson Cancer Center, Houston, TX USA; 2 Mallinckrodt Institute of Radiology, Washington University School of Medicine, St. Louis, MO USA; 3 Department of Nuclear Medicine, The University of Texas MD Anderson Cancer Center, Houston, USA; 4 Department of Radiation Physics, The University of Texas MD Anderson Cancer Center, Houston, USA

**Keywords:** Positron emission tomography computed tomography, Attenuation correction, Neural networks, Deeplearning, Artificial intelligence, Data driven gating, Misregistration correction, Respiratory motion

## Abstract

**Purpose:**

Misregistration between CT and PET can result in mis-localization and inaccurate quantification of the tracer uptake in PET. Data-driven gated (DDG) CT can correct registration and quantification but requires a radiation dose of 1.3 mSv and 1 min of acquisition time. AI registration (AIR) does not require an additional CT and has been validated to improve registration and reduce the ‘banana’ misregistration artifacts around the diaphragm. We aimed to compare a validated AIR and DDG CT in registration and quantification of avid thoracic lesions misregistered in DDG PET scans.

**Methods:**

Thirty PET/CT patient data (23 with ^18^F-FDG, 4 with ^68^Ga-Dotatate, and 3 with ^18^F-PSMA piflufolastat) with at least one misregistered avid lesion in the thorax were recruited. Patient studies were conducted using DDG CT to correct misregistration with DDG PET data of the phases 30 to 80% on GE Discovery MI PET/CT scanners. Non-attenuation correction DDG PET and misregistered CT were input to AIR and the AIR-corrected CT data were output to register and quantify the DDG PET data. Registration and quantification of lesion SUV_max_ and signal-to-background ratio (SBR) of the lesion SUV_max_ to the 2-cm background mean SUV were compared for each of the 51 avid lesions.

**Results:**

DDG CT outperformed AIR in misregistration correction and quantification of avid thoracic lesions (1.16 ± 0.45 cm). Most lesions (46/51, 90%) showed improved registration from DDG CT relative to AIR, with 10% (5/51) being similar between AIR and DDG CT. The lesions in the baseline CT were an average of 2.06 ± 1.0 cm from their corresponding lesions in the DDG CT, while those in the AIR CT were an average of 0.97 ± 0.54 cm away. AIR significantly improved lesion registration compared to the baseline CT (*P* < 0.0001). SUV_max_ increased by 18.1 ± 15.3% with AIR, but a statistically significantly larger increase of 34.4 ± 25.4% was observed with DDG CT (*P* < 0.0001). A statistically significant increase in SBR was also observed, rising from 10.5 ± 12.1% of AIR to 21.1 ± 20.5% of DDG CT (*P* < 0.0001). Many registration improvements by AIR were still left with misregistration. AIR could mis-localize a lymph node to the lung parenchyma or the ribs, and could also mis-localize a lung nodule to the left atrium. AIR could also distort the rib cage and the circular shape of the aorta cross section.

**Conclusions:**

DDG CT outperformed AIR in both localization and quantification of the thoracic avid lesions. AIR improved registration of the misregistered PET/CT. Registered lymph nodes could be falsely misregistered by AIR. AIR-induced distortion of the rib cage can also negatively impact image quality. Further research on AIR’s accuracy in modeling true patient respiratory motion without introducing new misregistration or anatomical distortion is warranted.

Whole-body (WB) PET/CT is a comprehensive technology for structural, functional, and molecular phenotyping of cancer at the WB level. It is a standard imaging tool for managing cancer patients for surgery, radiotherapy, chemotherapy, or a combination of these treatments [1]. (WB) PET/CT is normally conducted with a free-breathing (FB) CT followed by a FB PET. No breathing instructions are given to the patient. Misregistration between CT and PET is common and can result in mislocalization and inaccurate quantification of the tracer uptake in PET [2]. The extent of PET quantitation errors in the region of misregistration depends on the degree of misregistration between PET and CT and the distribution of radiotracer surrounding the misregistration area [3]. Although misregistration is often identified near the diaphragm, it can also occur in any part of the thorax and the abdomen. Misregistration could mistake a true positive for a false negative response [4]. It could also induce artifactual myocardial defects in 40% of cardiac PET/CT imaging [5].

Average CT, which is averaged from the cine CT images acquired at the gantry rotation of less than 1 s over a respiratory cycle, is an effective solution to improve the registration of CT and PET [6]. Average CT has a similar temporal resolution as PET, which is averaged from many breath cycles. However, it may not register well with motion-free data-driven gated (DDG) PET, which has gained popularity in the clinic for its effectiveness in motion correction and simplicity of use [7]. Breath-hold (BH) at normal expiration during CT acquisition could improve the registration between CT and PET [8]. However, it typically requires coaching the patient to BH with a respiratory monitoring device, which is difficult to perform in the clinic [9]. Coaching a patient to BH at end-expiration (EE) or deep-expiration without a respiratory monitoring device has not shown clinical benefits when compared to normal FB without coaching [10, 11].

DDG CT, which is derived from the change of the lung density and the body circumference of the cine CT images over a respiratory cycle, has been shown to register well with DDG PET [12]. DDG CT and DDG PET do not need a respiratory monitoring device and can be applied to any WB PET/CT scan [13]. Average CT and DDG CT can be used for attenuation correction of PET and DDG PET, respectively [12]. We have implemented DDG CT to combat misregistration artifacts in our clinic [12]. It requires a radiation dose of 1.3 mSv and 1 min of cine CT acquisition [13]. Compared to a repeat PET/CT scan over regions with PET/CT misregistration, DDG CT saved 65% of the CT radiation dose and saved scan time to avoid a repeat PET acquisition [13].

Deep-learning artificial intelligence (AI) for improving PET AC by elastic registration of the CT is a promising technique to reduce misregistration artifacts in PET/CT caused by respiratory, gross voluntary motion, or both [14, 15]. The feasibility of AI registration (AIR) has been demonstrated in WB PET/CT on multiple tracers and myocardial perfusion imaging at low-count statistics [15]. Conventional registration methods have generally been built on deformable image registrations that maximize some predefined image similarity metric iteratively [16, 17]. They are computationally expensive, sensitive to image noise, and generally unreliable for the deformable registration of anatomical CT data to match with functional PET data in PET/CT [14].

One promising AIR is a computational neural network (CNN) trained in a supervised fashion for the registration task. It does not rely on the pre-aligned pair of inter-modality images generally required during training [18], or complex similarity metrics [19]. This method has been termed AIR-PET as an AI registration tool for PET/CT [10, 20]. It has two distinct modules: a feature extractor and a displacement vector field (DVF). It combines non-AC PET and misregistered CT data and returns the relative DVF between them. The CNN was trained in a supervised fashion using simulated inter-image motion. The DVF was used to resample the CT image volumes, elastically warping them to match the corresponding PET distributions spatially [15]. The AIR method opens the possibility for physiologically gated or dynamically acquired PET data because it is not feasible for a single CT to have accurate AC for every frame of dynamic PET data [15]. The AIR method overcomes a major challenge in inter-modality PET and CT registration, as their voxel intensities are not correlated [14].

The training and validation of AIR included 22 WB PET/CT datasets of ^18^F-FDG (15), ^68^Ga-PSMA (5), and ^68^Ga-Dotatoc (2) with alignment; 10 were used for training and 12 for validation [15]. The smaller sub-volumes were extracted from the larger image data sets to serve as the network inputs to produce a large pool of augmented training patch data, which were separately deformed using random combinations of translation (0 ± 15 mm), rotation (0 ± 11.25^0^), and anisotropic voxel scaling (1 ± 0.1). Low-frequency, normal random tensor fields were added to simulate additional elastic effects. The registration for a WB PET volume takes less than a minute with a graphic processing unit (GPU) and could potentially be incorporated into the standard clinical PET/CT workflow. AIR improved the registration and image quality of WB PET/CT for 30 patients from six anatomic sites chosen for their susceptibility to PET/CT misregistration artifacts (superior liver margin, inferior liver margin, superior splenic margin, inferior splenic margin, right kidney, and left kidney) [15, 20]. The analysis also demonstrated improved uniformity of the liver and signal-to-background ratio (SBR) of superior hepatic margin volume of interest to inferior lung volume of interest [20]. AIR also significantly reduced the ‘banana’ artifacts around the diaphragm and improved the SBR around the diaphragm [10]. It consistently led to equivalent or improved PET image quality relative to the misregistered CT [10]. However, the assessments were only made with the misregistered and AIR-corrected PET/CT data and were limited by a lack of avid lesions impacted by misregistration and misregistration-corrected PET/CT data [10, 20].

To gain a better understanding of the potential benefits and limitations of misregistration correction with AIR, we aimed to evaluate motion-free avid lesions of DDG PET in patient studies that were identified to have a misregistration artifact and were misregistration corrected by both AIR CT and DDG CT [2, 12].

## Methods and materials

Thirty WB PET/CT patient data with at least one avid lesion in the thorax impacted by misregistration were retrospectively recruited to compare AIR and DDG CT for misregistration correction. The selection of the patients was sequential in time. The 30 studies included three radiotracers: 23 with ^18^F-FDG, 4 with ^68^Ga-Dotatate, and 3 with ^18^F-PSMA piflufolastat. In comparison, there were 22 studies of ^18^F-FDG, ^68^Ga-PSMA, and ^68^Ga-Dotatoc used in the training and validation of AIR. The targeted injected activities were 370, 185, and 370 MBq for ^18^F-FDG, ^68^Ga-Dotatate, and ^18^F-PSMA piflufolastat, respectively. The uptake time was 60 min. The patient studies were collected in a routine clinical procedure. A low-dose cine CT scan, covering a misregistered area of approximately 14 cm, was acquired after the PET scan to correct the misregistration [13]. The decision to correct for misregistration artifacts was made by the technologist after an image review of the thorax and the abdomen during the acquisition of the last bed position (normally at the mid-thigh) of the WB PET/CT scan [13]. The institution’s ethics review board approved the study. No patient consent was needed as this was a retrospective study.

The data were collected on the GE Discovery MI 25-cm axial field-of-view (AFOV) PET/CT scanners [21]. The FB WB PET/CT scan protocols were:

1. Helical CT scan of 120 kVp, 0.984 pitch, 0.5 s gantry rotation time, 64 × 0.625 mm x-ray collimation, tube current modulation (TCM) based on anterior to posterior scout view, noise index = 30, maximum mA = 560 and minimum mA = 60 and 100 for without and with injection of iodinated contrast, respectively. For iodinated contrast injection, the CT scan would start 56 s after injection of 100 cc of iohexol (Omnipaque-300) or iodixanol (Visipaque-320);
2. PET scan of acquisition time per bed position of 2 min for the torso of body mass index (BMI) < 35, 2.5 min for BMIs ≥ 35 but < 40, and 3 min for BMIs ≥ 40. The overlap between two consecutive bed positions was 25 slices or 28%. When the scan reached the legs, acquisition time was reduced to 1.5, 2, and 2 min for BMIs of < 35, 35 to 40, and ≥ 40, respectively;
3. Low dose cine CT scan of less than 1 min acquisition time for about 14 cm coverage over the misregistration area was acquired for 5 s per 2 cm scan position [13]. The cine CT protocol was 120 kVp, 0.8-sec gantry rotation time, 0.7-sec image reconstruction interval, 8 × 2.5 mm x-ray collimation, TCM of noise index = 70, maximum mA = 20, and minimum mA = 10. The 5-second CT duration was selected to cover 97.5% of the normal respiration rates of patients aged at least 65 [22]. Cine CT data was collected during the patient’s transition from PET to CT, before release. No breathing instructions were given to the patient in the PET/CT or cine CT scan.

The cine CT data was sent to a server for DDG CT processing. The patient was discharged. The data transfer and processing time for the DDG CT data was approximately 3 min. The DDG CT was sent back to the server and was used for AC of the DDG PET between 30 and 80% of the respiratory cycle to avoid image blurring. The correction was typically performed during the acquisition of the first one to two-bed acquisitions of the data of the next patient. Non-AC DDG PET data was paired with misregistered CT data and processed by the AIR to generate an AIR CT for AC of the DDG PET data, which was derived from the quiescent breathing phases of 30 to 80%.

All DDG PET images were reconstructed with the ordered subset expectation-maximization with 2-iterations and 17 subsets (VPFX-S), including time-of-flight (VUE Point FX), image recovery (SharpIR), and a Standard Z-filter with a post-reconstruction filtering of 5 mm filter cutoff. The various PET reconstructions differed only in the attenuation map used: baseline misregistered CT, AIR CT, or DDG CT.

For each patient, up to three lesions were selected for analysis, and lesion quantification and PET/CT registration were compared. The criterion for correct registration was complete overlap between radiotracer activity in PET and the corresponding soft tissue (lung nodule or lymph node) visualized on CT. Lesion SUV_max_ and SBR were measured on the PET data with the MIM Software 7.1.4 (MIM Software Inc., Cleveland, Ohio, USA), with the background defined as the mean SUV in a 2 cm diameter near the lesion. The distances between lesions on baseline and AIR images relative to corresponding lesions on DDG CT, as well as the lesion SUV_max_ and SBR values of AIR CT and DDG CT relative to baseline CT, were compared using either a two-tailed paired t-test (for normally distributed differences, as determined by the Shapiro–Wilk test) or a two-tailed Wilcoxon signed-rank test (for non-normally distributed differences). All statistics were calculated with GraphPad Prism 10.3.1 (GraphPad Software, San Diego, California, USA).

## Results

There were 51 avid thoracic lesions in the 30 patients. Misregistration correction by DDG CT was accurate for all 51 lesions. All the lesions were near the diaphragm. The lesion sizes were 1.16 ± 0.45 (mean ± standard deviation) cm. Relative to AIR CT, DDG CT improved lesion registration between DDG PET and CT in 90% (46/51) of the lesions, with the remaining 10% (5/51) being similar between AIR CT and DDG CT. The lesions in the baseline CT were an average of 2.06 ± 1.0 cm from their corresponding lesions in the DDG CT, while those in the AIR CT were an average of 0.97 ± 0.54 cm away. AIR CT significantly improved lesion registration compared to the baseline CT, based on the two-tailed paired t-test (*P* < 0.0001). AIR CT frequently improved registration by elevating the diaphragm position from inspiration to expiration. However, misregistration persisted for most lesions. Inferior-to-superior movement of the rib cage was frequently observed when comparing baseline CT to AIR CT, but not when comparing baseline CT to DDG CT. A lymph node may be displaced into the lung parenchyma mimicking a lung nodule or into the bones mimicking a bone metastasis, and a lung nodule could be displaced into the left atrium of the heart. Distortions of the vertebral body and the circular shape of the aorta cross-section were also observed. %SUV_max_ increases for the lesions from baseline to AIR were 18.1 ± 15.3% and from baseline to DDG CT were 34.4 ± 25.4%. There was a statistically significant %SUV_max_ increase from AIR CT to DDG CT (*P* < 0.0001). SBR increases from baseline to AIR were 10.5 ± 12.1% and from baseline to DDG CT were 21.1 ± 20.5%. There was also a statistically significant SBR increase from AIR CT to DDG CT (*P* < 0.0001). Increased SUV_max_ generally correlated with improved lesion registration between CT and PET, especially for lung lesions [13]. SUV_max_ decreased after improved registration for 3 lesions with AIR and 1 lesion with DDG CT.

**Fig. 1:**
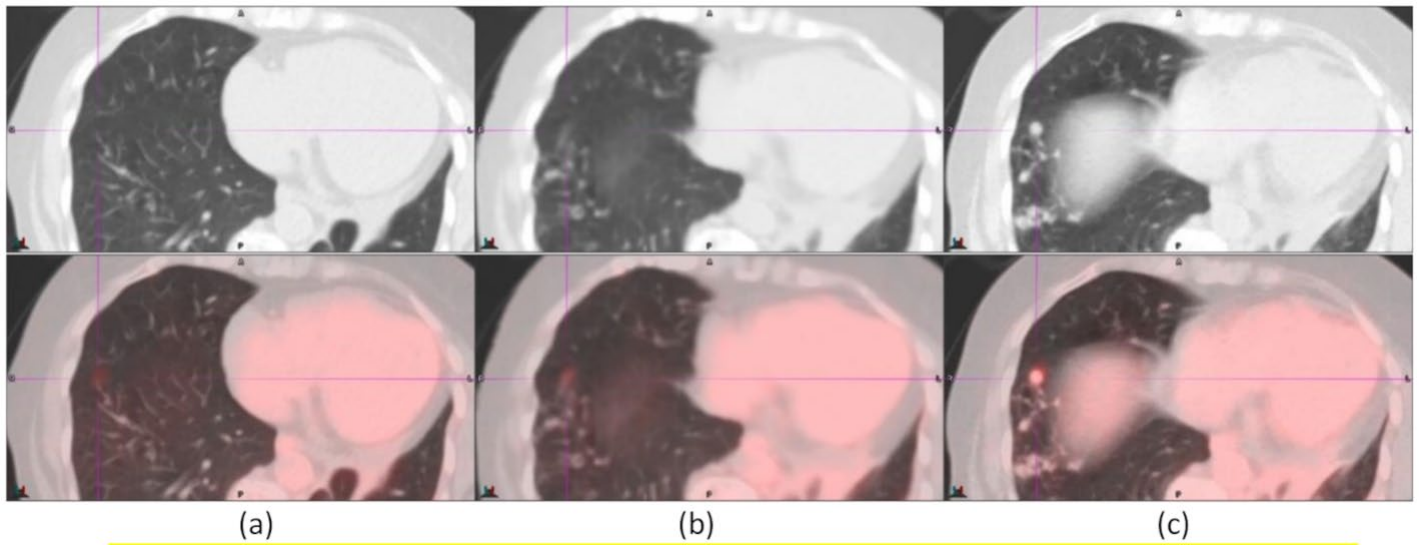
A 0.9-cm lung nodule was registered in DDG CT (**c**) but not in baseline (**a**) or AIR (**b**). The upper panel is CT, and the bottom panel is the fusion of PET and CT. The SUV_max_ values of the lung nodule were 1.87 for baseline, 2.08 for AIR and 4.21 for DDG CT

Figure 1 shows a typical improvement in the registration of an avid lesion from baseline (a) to AIR (b), and from AIR (b) and to DDG CT (c). The lesion was a 0.9-cm lung nodule in the right lower lobe of an ^18^F-FDG study. The CT (upper panel) and fusion of PET and CT (lower panel) demonstrated an improved registration of the diaphragm from baseline to AIR to DDG CT. The lung nodule was registered between CT and PET by DDG CT. The SUV_max_ values of the lung nodule were 1.87 for baseline, 2.08 for AIR, and 4.21 for DDG CT. Following misregistration correction by DDG CT, this lung nodule became avid (SUV_max_ ≥ 2.5), a finding not observed in baseline or AIR.

**Fig. 2:**
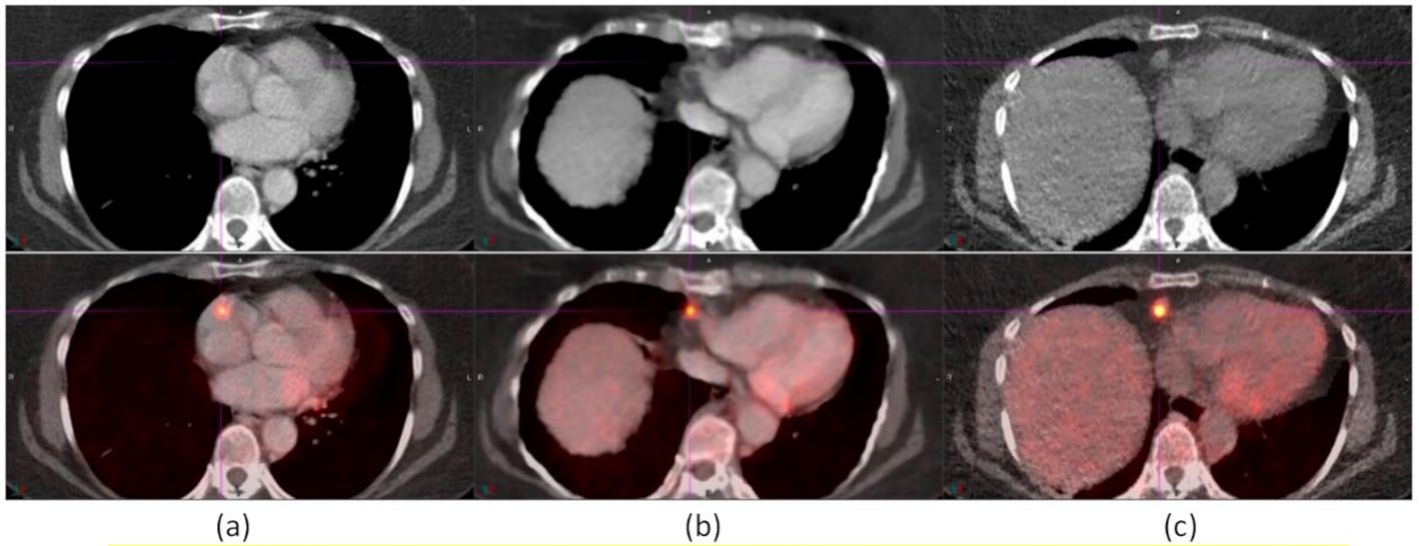
A 1.4-cm para-cardiac lymph node was registered with DDG CT (**c**) but not with baseline (**a**) or AIR (**b**). The SUV_max_ values of the lymph node were 10.78 for baseline, 12.43 for AIR, and 15.41 for DDG CT

The following studies illustrate the benefits and limitations of AIR. Figure 2 shows an improvement in the registration of a 1.4-cm para-cardiac lymph node from baseline to AIR, and from AIR and to DDG CT in an ^18^F-FDG study. The lymph node was mispositioned to the right atrium in baseline and its position was not clear due to the cardiac motion artifacts in AIR. On DDG CT, the lymph node was registered between CT and PET. The cross-sectional diaphragm region was notably larger on DDG CT than on AIR. The SUV_max_ values of the lymph node were 10.78 for baseline, 12.43 for AIR, and 15.41 for DDG CT. There was CT contrast in baseline and AIR but not in DDG CT. The shape of the vertebral body and the positions of the ribs in AIR were notably different from those in baseline or DDG CT.

**Fig. 3:**
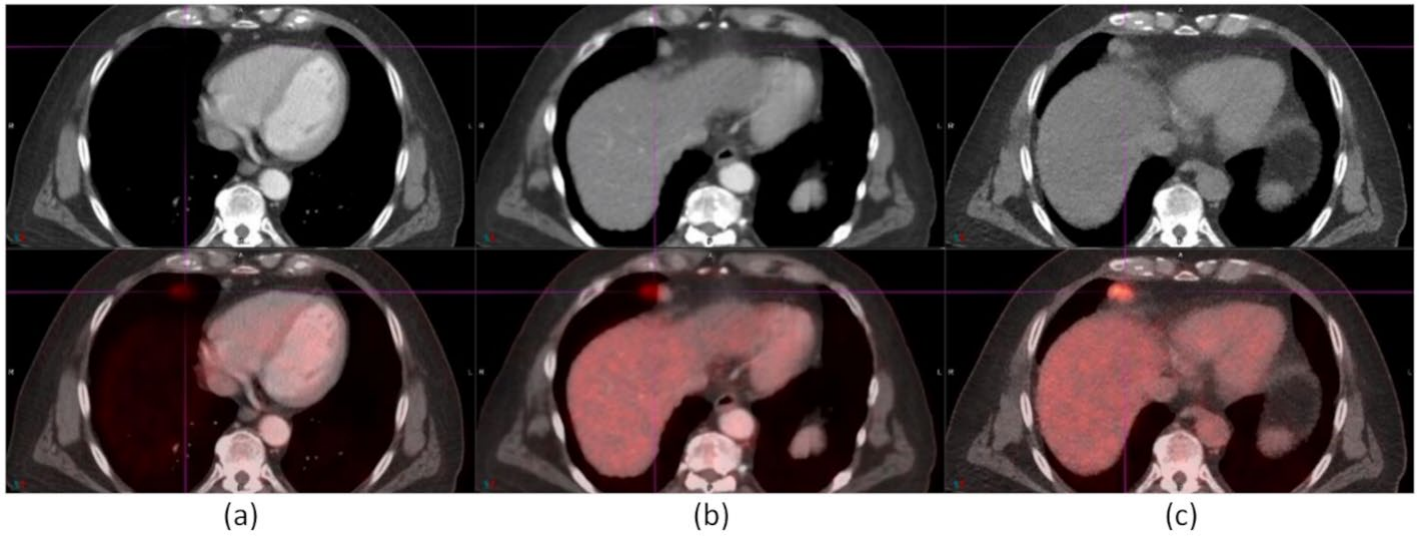
Two anterior diaphragmatic lymph nodes, measuring 1.0 cm and 1.2 cm, were identified on DDG CT (**c**) but were indistinguishable in both baseline (**a**) and AIR (**b**). The SUV_max_ values for these nodes were 3.73 (baseline) and 5.26 (AIR), to 7.18 and 6.37 on DDG CT. Notably, the shape of the vertebral body and the locations of the ribs in AIR (**b**) differed from those observed in baseline (**a**) or DDG CT (**c**)

Figure 3 shows two anterior diaphragmatic lymph nodes of sizes 1.0 and 1.2 cm in an ^18^F-FDG study not registered in baseline, partially registered in AIR, and well-registered in DDG CT. The two lymph nodes were only distinguishable in DDG CT but indistinguishable in baseline and AIR. The SUV_max_ values of the lymph nodes were 3.73, 5.26, and 7.18, for baseline, AIR, and DDG CT, respectively. The SUV_max_ value of the 2nd lymph node in DDG CT was 6.37. There was CT contrast in baseline and AIR but not in DDG CT. The shape of the vertebral body and the rib positions in AIR were notably different from those in baseline or DDG CT.

**Fig. 4:**
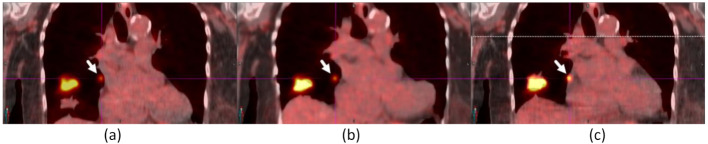
A 0.8-cm para-cardiac lymph node (indicated by an arrow) was registered in DDG CT (**c**), but mispositioned to the lung parenchyma in both baseline (**a**) and AIR (**b**). The SUV_max_ values for this lymph node were 4.76 (baseline), 5.01 (AIR), and 5.56 (DDG CT). Furthermore, a lung lesion located 3 cm above the diaphragm showed improved registration in both AIR and DDG CT. Its SUV_max_ values were 9.02 (baseline), 11.27 (AIR), and 10.64 (DDG CT)

Figure 4 illustrates improved registration of a 3-cm lung nodule with both AIR and DDG CT. However, a 0.8-cm para-cardiac lymph node (indicated by an arrow) was misregistered to the lung parenchyma in baseline and AIR but was registered in DDG CT in an ^18^F-FDG study. The image also displays a fusion of baseline CT and DDG CT, marked by a horizonal line on DDG CT. The area above this line was attenuation correction (AC) with baseline CT, while the area below was AC with DDG CT. This fusion is essential because AC requires CT scan coverage to be equal to or greater than PET scan coverage. The SUV_max_ values of the lung nodule (the lymph node) were 9.02 (4.76) for baseline, 11.27 (5.01) for AIR, and 10.64 (5.56) for DDG CT.

**Fig. 5:**
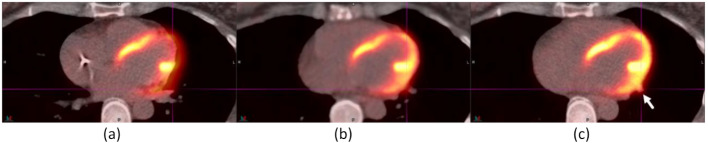
A 0.5-cm lung lesion (indicated by an arrow) was called in DDG CT (**c**) but not in baseline (**a**) or AIR (**b**). The SUV_max_ values of the lung nodule were 6.59 for baseline, 7.68 for AIR, and 7.96 for DDG CT

Figure 5 shows an additional lung lesion of size 0.5 cm in an ^18^F-FDG study, whose SUV_max_ value was 7.96, which was by the heart in DDG CT but not indicated in baseline or AIR even though there was an improved registration by AIR. This example suggested that motion correction and registration improvement by both DDG PET and DDG CT could improve the diagnosis.

**Fig. 6:**
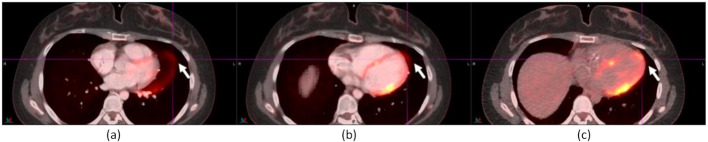
The anterior-lateral walls (indicated by an arrow) of the heart were registered in DDG CT (**c**), mostly registered in AIR (**b**), but significantly misregistered in baseline (**a**)

Figure 6 shows a misregistration of the heart in an ^18^F-FDG study, improved by AIR and registered in DDG CT. AIR still missed a portion of the anterior-lateral walls. Exercise caution when employing AIR for heart registration in cardiac imaging.

**Fig. 7:**
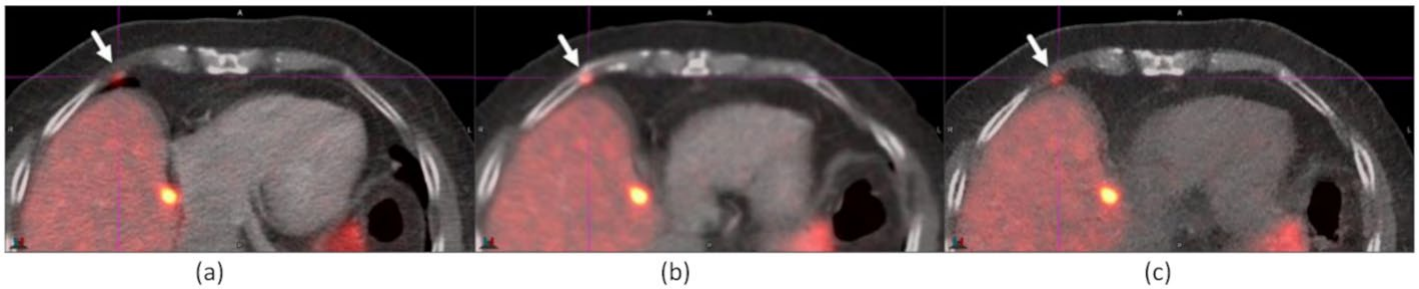
A 0.5-cm right supradiaphragmatic lymph node (indicated by an arrow) of a ^68^Ga-Dotatate study in baseline (**a**) and DDG CT (**c**) was mispositioned to the ribs in AIR (**b**). The SUV_max_ values were 8.62 for baseline, 9.19 for AIR, and 8.79 for DDG CT

Figure 7 shows a 0.5 cm right supradiaphragmatic lymph node in a ^68^Ga-Dotatate study. The lymph node with CT contrast was slightly misregistered in baseline, and the same lymph node after CT contrast washout was registered in DDG CT. AIR’s misregistration of this lymph node to the ribs created misleading information, potentially suggesting bone metastasis.

**Fig. 8:**
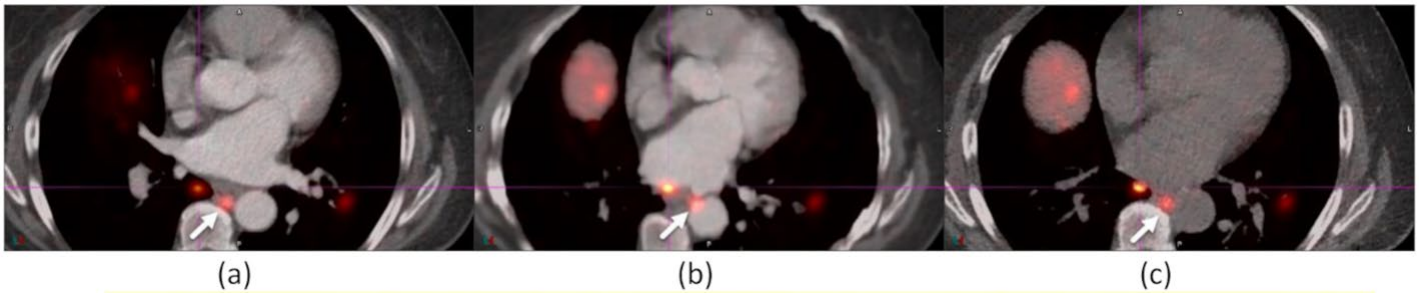
A 0.7-cm lung lesion at the crosshair in baseline (**a**) and DDG CT (**c**) was mispositioned to the left atrium of the heart in AIR (**b**). A separate lymph node (indicated by an arrow) by the vertebral body and the aorta in baseline and DDG CT was intruding the aorta, which was no longer circular in AIR. The SUV_max_ values of the lung lesion were 20.3 for baseline, 25.0 for AIR, and 22.6 for DDG CT

Figure 8 shows a lung lesion at the crosshair in an ^18^F-FDG study near the vertebral body was mispositioned to the left atrium in AIR. A separate lymph node by the vertebral body and the aorta in baseline and DDG CT appeared to intrude the aorta in AIR. AIR distorted the rib cage and transformed the normally circular aortic cross-section into a non-circular shape.

**Fig. 9:**
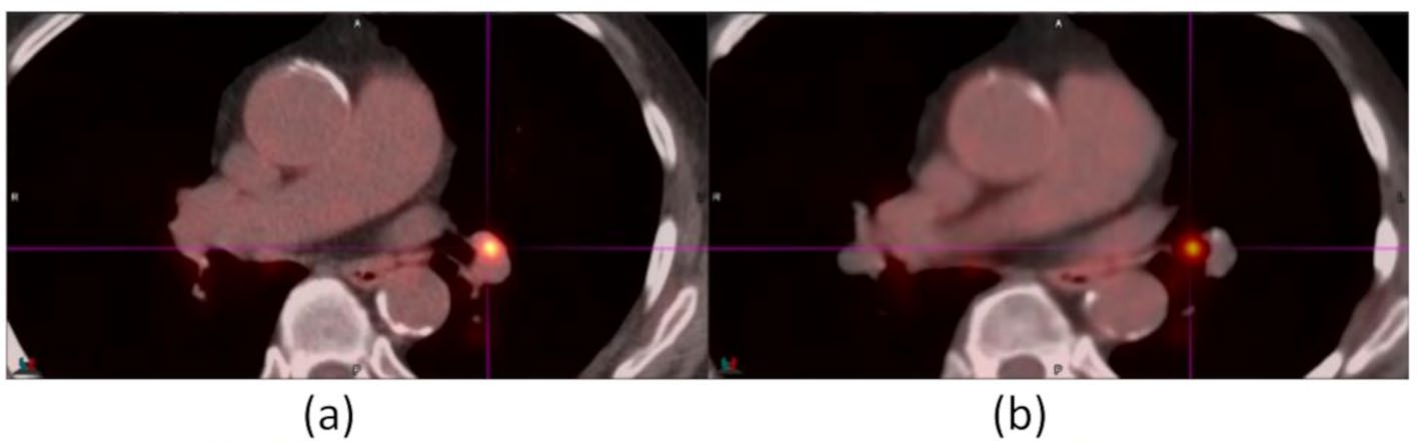
A 0.6-cm left infra-hilar lymph node was registered in baseline (**a**) but misregistered in AIR (**b**). The SUV_max_ values were 8.5 for baseline and 8.7 for AIR

Figure 9 shows a 0.6-cm left infra-hilar lymph node in an ^18^F-FDG study registered in baseline but misregistered in AIR. AIR could introduce a new misregistration for a registered lesion. There was no corresponding DDG CT for this registered lymph node, as cine CT acquisition was limited to the region of misregistration, which did not include this specific lymph node. This lymph node was also not one of the 51 lesions included in this study.

## Discussions

We compared a validated AIR method and DDG CT in the registration and quantification of avid thoracic lesions that were misregistered on DDG PET scans. In this study of 30 patients, all 51 lesions were registered between DDG CT and DDG PET. Although the diaphragm position improved from baseline to AIR, most lesions (46/51, 90%) remained misregistered after AIR. Only 10% (5/51) of the lesions maintained similar registration correction between AIR and DDG CT. An example that highlights this is the 0.8 cm para-cardiac lymph node still misregistered by AIR in Fig. 4. Most SUV_max_ increases agreed with the improved registration of the lesions between CT and PET. However, there were 3 lesions with AIR and 1 lesion with DDG CT where SUV_max_ decreased after improved registration. Although AIR improved registration and quantification of the lesions, we still observed a distinct difference when AIR and DDG were compared side-by-side. Most of the improvements achieved by AIR were partial, suggesting that the simulated respiratory motion modeled in AIR did not fully capture the extent of respiratory motion observed in patient studies. In our previous study, the diaphragm misregistration between PET and CT was found to exceed four times the diaphragmatic motion during normal respiration, as measured by the displacement from end-inspiration to end-expiration on cine CT [16].

CT contrast injection in 13 studies and 21 lesions increased SUV_max_ for the lesions in baseline and AIR but not DDG CT (Figs. 2, 3, 6 and 7, and 8). One study suggested an increase of SUV_max_ by 8.4% at a lesion when CT contrast was introduced [23]. Contrast injection in CT before the PET scan would not impact DDG CT as the cine CT was taken after the PET scan of ≥ 10 min acquisition (> 5 bed-positions and 2 min per bed-position). Any CT contrast would have been cleared before the PET scan. Non-contrast DDG CT should be more accurate than contrast-enhanced CT for quantitative PET metrics. However, this increase in SUV_max_ by contrast-enhanced CT in baseline and AIR did not alter the statistically significant increases in SUV_max_ or SBR from AIR to DDG CT.

The findings agreed with the results of the previous two studies that AIR improved the registration of PET and CT when it was compared to baseline PET/CT with misregistration [10, 20]. Image quality was improved in a reader’s evaluation and the uniformities of the liver, spleen, and kidneys, impacted by misregistration, were also enhanced by AIR [20]. Image quality and SBR were improved in a clinical impact cohort of 20 patient studies with a significant ‘banana’ artifact [10]. In a validation cohort of 20 patients (one regular FB CT and one BH CT at EE before PET), PET and DDG PET with AIR always scored at least as well as PET and DDG PET with either FB or BH CT, when matched to the most quiescent 35% of DDG PET data [10]. In one patient study, BH CT at EE was rated as ‘poor quality’ and in several patient studies, it was rated as ‘acceptable quality’. It was concluded that BH CT at EE performed worse than FB CT. The conclusion was that AIR never degraded image quality compared to FB CT or BH CT at EE [10]. Despite these prior results, in the present analysis, AIR did not perform as well as DDG CT for misregistration correction between DDG PET and CT.

The results of EE CT to match with DDG PET were inconsistent between this retrospective study, which used DDG CT derived from a FB cine CT after the PET scan, and the prospective study, which used a BH CT at EE before the PET scan [10]. DDG was better than AIR in this cohort of 30 patients and 51 avid lesions in registration, quantification, and SBR. On the other hand, AIR was better than FB CT and FB CT was better than BH CT at EE for a cohort of 20 patients in a visual quality blind assessment using a five-level Likert scale, and AIR had a higher SUV_max_ for the lesions when compared to either FB CT or BH CT at EE [10]. No breathing instruction was given to the patients in this study for the cine CT scan. However, acquiring BH CT at EE required coordination between the patient and the technologist who operates the PET/CT scanner. Some patients may succeed in following the breathing instructions, while others may not. Even the ones who follow the breathing instructions may not breathe to the level of EE to match with DDG PET. In addition, it adds complexity if a respiratory monitoring device is used during patient coaching, which needs additional hardware setup [9]. Miscommunication could also lead to unexpected results, such as BH CT at EE performing worse than FB CT when registering with DDG PET [10]. Not giving breathing instructions to the patient may be a better way of keeping the patient’s breathing normal [2]. Acquiring DDG CT to correct for misregistration is a standard of care procedure in our clinic [13]. It has been shown to improve registration in more than 99.5% of lesions in a study of 650 PET/CT studies with misregistration [24].

The finding that AIR could introduce additional misregistration on the already registered lymph nodes in Figs. 7, 8 and 9 was concerning. These lymph nodes were adjacent to the chest walls, the vertebra body, and the lung parenchyma. In addition, the distortion of the rib cage in many of our patient studies and the distortion of the circular shape of the aorta cross-section in Fig. 8 could degrade a reader’s confidence. These findings have not been reported before. Currently, AIR treats bones as any other soft tissue subject to elastic registration [15]. The rib cage expands and contracts with respiration. It may not fit well with the model of elastic registration. Measures to improve registration of the bones in AIR are warranted. Although AIR achieved the goal of reducing the misregistration ‘banana’ artifacts by the diaphragm, eliminating these new misregistration and distortion artifacts from AIR will be important for the acceptance of AIR as most of our patient studies are identified not just by the ‘banana’ artifacts but also by misregistration of avid lesions that are the focus of a diagnostic PET/CT scan.

There may be limitations to using AIR for misregistration correction of the PET/CT data. AIR relies on the deformation of misregistered CT data, which may have lost some granularity or detail due to deep inspiration during the CT scan and may still contain motion artifacts that are difficult to eliminate. AIR was trained with limited data sets (10 for training and 12 for validation) [15]. AIR seemed to correct the gross misregistration by the diaphragm well but still left most of the lesions misregistered between CT and PET. Figures 2, 3, 4, 5 and 6 were examples.

AIR was trained using simulated misregistered data derived from registered PET/CT scans, rather than actual misregistered and corrected PET/CT pairs. This is due to the general unavailability of such paired data in clinical practice [15]. DDG CT could potentially provide a source of misregistered and corrected PET/CT data for future AIR training.

This study has some limitations. First, the AIR was trained using data from one vendor and evaluated using data from another, potentially introducing a training bias that could make the AIR appear less favorable. Cine CT is not yet available on Siemens PET/CT scanners. However, it has become the scan mode for intelligent 4D-CT of Siemens CT scanners for radiation therapy treatment simulation and may be incorporated into Siemens PET/CT scanners in the future [25]. Second, the study’s single-institution design may limit the generalizability of its findings.

## Conclusions

DDG CT demonstrated superior performance to AIR in both misregistration correction and quantification of most avid thoracic lesions on DDG PET. Both quantification and signal-to-background ratios significantly increased from AIR to DDG CT. While AIR improved both localization and quantification of avid thoracic lesions, most corrections still exhibited residual misregistration. Furthermore, AIR sometimes falsely misregistered previously aligned lymph nodes and distorted the aorta and rib cage, potentially degrading image quality. Further research is needed to improve AIR’s accuracy in modeling respiratory motion without introducing new misregistration or anatomical distortion.

## Data Availability

The datasets generated during and/or analyzed during the current study are available from the corresponding author upon reasonable request.

## Declarations

### Disclaimer

This research was supported in part by R01HL157273-02 and R01-EB034692-01A1. The views expressed are solely those of the authors and do not reflect those of the National Institutes of Health, MD Anderson Cancer Center, or Washington University School of Medicine.
